# Psychometric properties of an instrument measuring monkeypox knowledge, perception, and beliefs of health threat in health science students in a middle-income country

**DOI:** 10.12688/f1000research.145169.3

**Published:** 2026-08-04

**Authors:** Oriana Rivera-Lozada, Miguel Ipanaqué-Zapata, Isabel Cristina Rivera-Lozada, Elvis Siprian Castro-Alzate, Hebe Hernández-Romero, Mariana Gómez-Rivera, César Antonio Bonilla-Asalde

**Affiliations:** 1 Universidad Señor de Sipán, Chiclayo, Peru; 2 Universidad del Cauca, Popayán, Cauca, Departamento Economía, Colombia; 3 Universidad del Valle, Cali, Valle del Cauca, Programa Académico de Terapia Ocupacional, Colombia; 4 Universidad Santiago de Cali, Cali, Valle del Cauca, Colombia; 5 Pontificia Universidad Javeriana, Cali, Valle del Cacua, Colombia; 6 Hospital Daniel Alcides Carrión, Callao, Peru

**Keywords:** Monkeypox; Knowledge; Perception; Beliefs

## Abstract

**Objective:**

This study aimed to establish the factor structure and reliability of the evaluation instrument measuring monkeypox (Mpox) knowledge, perception and beliefs of health threat in students of the health area in two universities of Peru during 2022.

**Methods:**

The methodology used was psychometric in nature. The study variable on knowledge of Mpox was assessed using a 17-item questionnaire developed by Ricco et al., originally applied to health professionals, adapted to Peruvian context, and administered to 416 students.

**Results:**

The results showed adequate goodness-of-fit indicators, with RMSEA and SRMR values below 0.08 and a TLI above 0.90, confirming an acceptable model fit. Reliability was adequate for knowledge of Mpox (KR20 = 0.70 and α = 0.73), with perception of health threats showing the highest reliability (α = 0.88 and Ω = 0.89).

**Conclusions:**

The instrument demonstrated an adequate factor structure and acceptable reliability for measuring Mpox knowledge, perception, and health threat beliefs in Peruvian health students, supporting its applicability in future research and public health interventions.

## 1. Introduction

The disease caused by the monkeypox (Mpox) is considered, after the eradication of smallpox in 1979, the most important infection caused by a virus of the Poxviridae family of the Orthopoxvirus genus. ^1^^,^^2^ During the period 2017–2021, outbreaks of cases were reported in Nigeria and other regions that received travelers from this origin. The last outbreak of cases recorded occurred in May 2022 in the United Kingdom, during the COVID-19 virus pandemic. ^3^ In July 2022, Mpox was declared a public health emergency of international concern by the World Health Organization due to the simultaneous record of cases in more than 70 countries. ^4^

Manifestations of the disease range from fever, lymphadenopathy, and a typical skin rash to systemic symptoms or even death, ^5^ which is self-limiting in most cases. However, there are more severe forms associated with immunosuppressed populations and vulnerable groups of children, pregnant women, and older men. ^6^^,^^7^ Mpox was initially considered a zoonotic disease; however, it currently incorporates contagion through human-human contact, where the main transmission route is direct contact between mucous membranes and skin in sexual activity, ^8^ and secondary routes are respiratory droplets and vertical transmission. ^8^^,^^9^

There have been 86,173 cases and 89 deaths confirmed in the world until February 24, 2023. The Americas accounts for 86% of the cases and includes six of the ten countries with the highest number of confirmed cases globally. ^10^ In the case of Peru, it is in the fourth place and accounts for 17% of the deaths caused by Mpox. ^11^^,^^12^ Compared to 2022, a significant decrease in cases was observed in 2023 in Latin America, in contrast to other regions of the world. In Latin America, concern about Mpox remains latent since, in addition to the frequency of cases, there are barriers such as social inequality, misinformation, a shortage of human and health resources, and political crises. ^13^

One aspect of interest is related to the level of knowledge of health personnel concerning Mpox, ^14^ where health professionals in training become relevant in outbreak contexts. For example, we can mention similar cases such as what occurred with the COVID-19 pandemic, which has highlighted the role of learning during university life, proposing strategies for a preventive approach to the healthcare profession, and providing bases for managing the perception of possible contagion. ^15^

The re-emergence of the disease caused by the Mpox generated epidemiological alerts worldwide and required health services to implement effective responses that guarantee effective prevention strategies based on mastering the conditions that led to the current state of this problem. ^16^^,^^17^ This situation invites us to explore the degree of knowledge as well as the characteristics, attitudes, perceptions and beliefs that influence the practices present during the interaction with users of health services.

Previous research has shown that health students have insufficient knowledge and inadequate attitudes towards Mpox. ^18^^–^^20^ A multicentric study conducted at three universities in the United Arab Emirates, Egypt, and Iraq evaluated the knowledge, attitudes, concerns, and mental health needs regarding Mpox among nursing students. Using a questionnaire with acceptable internal consistency (Cronbach’s alpha of 0.85), it was found that students, particularly in Egypt, have significant gaps in their knowledge and generally neutral or negative attitudes towards Mpox. Many experience moderate anxiety and perceive a considerable need for mental health support. Additionally, a positive correlation was observed between knowledge and attitudes, with significant variations based on nationality, educational level, and exposure to information, highlighting the need for personalized educational and support programs. ^19^ This underscores the importance of having appropriate tools to measure students’ knowledge, attitudes, and perceptions to promote quality health education and strengthen students’ skills in facing emerging health challenges. ^21^

The stated situation added to the Peruvian context in contrast to the literature review, reveals a limited number of psychometric studies on instruments that evaluate the knowledge, attitudes, and perceptions of health students regarding Mpox. ^22^ This motivated the study to establish the factor structure and reliability of evaluating knowledge, perceptions, and beliefs about health threats among health students concerning Mpox infection. Through the review and validation of the psychometric properties of this instrument, the aim is to promote high-quality education in health sciences that not only stays current with medical knowledge but also accurately reflects students’ perceptions and readiness to address health challenges, such as Mpox and other epidemiological threats.

## 2. Methods

### 2.1 Study design and scope

This research is a psychometric study carried out in two universities in the capital of Peru during the months of August to December 2022.

### 2.2 Population

A total of 436 health sciences students enrolled in the universities during the period of interest for this research were surveyed using non-probability convenience sampling. The inclusion criteria were as follows: being enrolled at the time of instrument administration, being over 18 years of age, voluntarily accepting informed consent, and having completed at least 50% of the questionnaires. Twenty of these students (4.5%) did not answer more than 50% of the questionnaires; for this reason, they were excluded from the study, which meant a final sample of 416 students.

### 2.3 Variables and instruments

The study variable on knowledge of Mpox was taken from the instrument created by Ricco et al. ^20^ and administered to health sciences students. From the original instrument, 17 items on knowledge of Mpox were extracted, organized into three response categories: “True”, “False” and “Don’t know”. The questions have a correct and incorrect question structure; for this reason, after collection, they were transformed into dichotomous items (correct answer=1 point and incorrect answer=0 points). The total score of the questionnaire ranges from 0 to 17 points. Following the recommendations of Lira and Caballero, ^23^ it was specified that the Spanish translation of the questionnaires was carried out by one translator and two professionals with expertise in the medical field, with the latter being members of the research team, in order to ensure alignment with the study’s objectives. Additionally, a back-translation process was implemented, in which one translator reviewed the translated version, and the final version was evaluated by a panel of three experts with qualitative content evaluation to confirm coherence and cultural appropriateness within the Peruvian context.

Regarding the variable of perception and belief of health threats, the instrument from Ricco et al.’s study ^20^ was also used. It comprised 11 items presented on a Likert scale with five response categories (1=strongly disagree, 5=strongly agree), while the items of threat to health due to other diseases transmitted through the virus used a threat perception magnitude scale with ten response categories reduced to five (1=least threat perception, 2=maximum threat perception), with a total score between 11 and 55 points.

### 2.4 Procedure

The instrument was validated by Ricco et al. (2022) ^20^ and adjusted to Peru through a pilot test that allowed irrelevant practices to be excluded. The collection of information received the approval of the university authorities and the informed consent of the students. The students were invited to participate voluntarily and contacted by email and WhatsApp to access the instrument through a Google form.

### 2.5 Data analysis

The psychometric analysis of the study was exploratory, using the weighted least squares means and variance-adjusted estimation (WLMSV) and polychoric matrix, as well as oblique rotation (quatrimin) to obtain the factor loadings of the dimensions of the study instruments.

This estimator minimises the discrepancy between the sample covariance matrix ( *s*) and the model-implied covariance matrix ( *σ*( *θ*)) as a function of the parameters *θ*:

$$F_{\text{WLMSV}}={\left(s-\sigma \left(\theta \right)\right)}^{\prime }W^{-1}\left(s-\sigma \left(\theta \right)\right)$$

Where *W* is the inverse of the asymptotic covariance matrix of *s*. ^24^ WLSMV employs robust standard errors and a mean–variance adjusted χ ^2^ test statistic. ^25, 26^ For ordinal variables, polychoric correlations were computed, defined as the Pearson correlation between continuous latent variables underlying the observed ordinal responses. ^26, 27^ Finally, an oblique quartimin rotation was applied to enhance factor interpretability.

Additionally, the explained variance was calculated as the ratio of the sum of the eigenvalues of the retained factors to the total eigenvalues, expressed as a percentage. Values above 60% are generally considered acceptable in psychometric studies. ^28^ Bartlett’s sphericity was also evaluated to determine if there is sufficient correlation between the variables and the Kaiser-Meyer-Olkin (KMO) adequacy index, which provides a measure of the adequacy of the data for factor analysis.

After that, the confirmatory factor analysis was obtained to show the goodness-of-fit indicators. The indicators considered were the Tucker-Lewis index (TLI), appropriate when it is greater than or equal to 0.90 ^28^; the standardized root mean squared residual index (SRMR) and the root mean square errors of approximation (RMSEA), appropriate when they are lower than or equal to 0.08. ^28^

Finally, the reliability of the instrument was obtained through the KR-20, Cronbach’s Alpha and Omega indicators, with values greater than 0.50, which are acceptable. R Studio software was used for the analyses of psychometric properties, along with the“lavaan”,“semTools” and “semPlot” packages (Rstudio ^®^, Boston, MA, USA). whose license belongs to the Universidad del Cauca.

### 2.6 Ethical principles

The students who participated in the study did so voluntarily and gave their informed consent in the research instrument form (questionnaire).

The research considered the Declaration of Helsinki and the protocol was approved by the ethics committee of the Norbert Wiener University, file 1787-2022. dated October 2022 where the investigation has been considered a minimal risk investigation.

## 3. Results

The main characteristics of the 416 study participants were identified, showing that the majority were women (78.61%), belonging to the age group of 18 to 20 years (39.90%), second-year students (48.32%), nursing professionals (61.78%), and attending a private university (90.87%) ( Table 1).

**Table 1.** Characteristics of the study sample (n=416).

Variables	n	%
**Gender**		
Female	327	78.61
Male	89	21.39
**Age**		
18-20	166	39.9
21-25	130	31.25
over 25	120	28.85
**Profession**		
Medicine	60	14.42
Nursing	257	61.78
Dentistry	99	23.8
**Type of University**		
Private	378	90.87
Public	38	9.13
**Year of study**		
1	18	4.33
2	201	48.32
3	37	8.89
4	65	15.63
5 and 6	95	22.83

Regarding the exploratory analysis of the study questionnaires, the polychoric matrix and the weighted least squares means and variance adjusted (WLMSV) method were used, adjusting the data for a robust interpretation of the relationships between the questionnaire items. It was observed that the factor loadings for all items exceeded the 0.40 threshold, ensuring reliable representativeness of each dimension within the evaluated constructs. For the Mpox knowledge questionnaire, the items with the highest factor loadings were: knowledge of Mpox transmission through direct contact (0.76) and respiratory secretions (0.73), as well as knowledge that infections are commonly associated with typical skin lesions (0.75). This indicates that these aspects best reflect the knowledge construct of Mpox. In terms of the perception and beliefs questionnaire, the items that most accurately captured the construct were: perception of the severity of Mpox infection (0.93) and the belief that an infection would affect one’s daily activities (0.87). These results suggest that participants tend to associate Mpox with significant impacts on daily life and perceive it as a serious threat. Additionally, the explained variance for the knowledge and perception and beliefs questionnaires on Mpox was high, reaching 84.6% and 87.1%, respectively, indicating that the items adequately represent each construct’s dimensions. The Kaiser-Meyer-Olkin (KMO) measure of sampling adequacy for both questionnaires was excellent, with values of 0.93 and 0.95, exceeding the recommended limit of 0.60. Furthermore, Bartlett’s test of sphericity was significant (p<0.05), confirming that the correlation matrix is suitable for factor analysis ( Table 2).

**Table 2.** Factor Loadings of the exploratory analysis for the instruments of knowledge, perception and beliefs about Mpox and diseases transmitted through viruses.

	Knowledge, perception and beliefs			
	D _1_^+^(λ)	D _1_^++^(λ)	D _2_^++^(λ)	D _3_^++^(λ)
**Variable 1: Knowledge about Mpox**				
Monkeypox (MPXV) is caused by a newly discovered virus.	0.72			
The MPXV virus circulates only among primates, including humans.	0.68			
In most cases, the MPXV virus evolves into an influenza-like illness without complications.	0.62			
MPXV infections are associated with typical skin lesions.	0.75			
Until recently, cases of MPXV have been mainly associated with traveling abroad.	0.71			
There is no effective vaccine against MPXV available to date.	0.67			
Effective drugs against MPXV are not available to date.	0.58			
MPXV can be transmitted through respiratory secretions.	0.73			
MPXV can be transmitted through saliva droplets.	0.7			
MPXV can be transmitted by direct contact.	0.76			
MPXV can be transmitted through body fluids.	0.74			
MPXV infection is associated with a high rate of systemic complications.	0.69			
MPXV causes less severe disease in children (age < iiv years) than in adults.	0.6			
The skin rash associated with MPXV is usually asymptomatic.	0.67			
Standard preventive measures are effective in preventing MPXV infection.	0.55			
Surface tension and the profusion of skin lesions associated with MPXV have prognostic value.	0.66			
MPXV is capable of surviving for several days on contaminated surfaces.	0.68			
**Variable 2: Perception and Beliefs about Mpox**				
Based on your current understanding, how do you perceive MPXV infection in the country? Regarding its severity		0.93		
Based on your current understanding, how do you perceive MPXV infection in the country? [Regarding its frequency]		0.82		
How would you rate the health threat posed by the following diseases? [monkeypox virus]			0.81	
How would you rate the health threat posed by the following diseases? [SARS-COv-1]			0.87	
How would you rate the health threat posed by the following diseases? [Tuberculosis]			0.83	
How would you rate the health threat posed by the following diseases? [Seasonal flu]			0.62	
How would you rate the health threat posed by the following diseases? [Hepatitis B virus]			0.84	
How would you rate the health threat posed by the following diseases? [Human immunodeficiency virus (HIV)]			0.80	
From your point of view, in the next 12 months, will the MPXV be a likely occurrence during daily activities?				0.87
From your point of view, in the next 12 months, will the MPXV significantly affect your daily activities?				0.87
From your point of view, in the next 12 months, are you confident you can recognize cases of monkeypox during your academic activity?				0.64
Explained Variance (%)	84.6	87.1		
KMO	0.93	0.95		
Bartlett sphericity (p-value)	0.037	0.021		

^+^D _1_: Knowledge dimension on Mpox, ^++^D _1_: Mpox country infection perception dimension, D _2_: Perception of threat from Mpox and other viral Infectious diseases, D _3_: Belief in the Impact of Mpox Transmission.

Regarding the confirmatory factor analysis, goodness-of-fit indicators were evaluated for each of the study questionnaires to determine how well the data fit the proposed model structures. Appropriate thresholds for these indicators were set as follows: RMSEA and SRMR values below 0.08 indicate a good model fit, while TLI values of 0.90 or above are also indicative of a well-fitting model. For the Mpox knowledge questionnaire, the goodness-of-fit indicators met these standards, with RMSEA and SRMR values of 0.065 and 0.075, respectively, both comfortably below the threshold. The TLI value of 0.92 further confirmed that the model fit was acceptable. These results suggest that the questionnaire reliably captures the knowledge dimension as designed. Similarly, the questionnaire assessing perceptions and beliefs about health threats, specifically those posed by Mpox and other viral diseases, demonstrated strong goodness-of-fit indicators. Both RMSEA and SRMR values were below 0.08, and the TLI was exceptionally high at 0.97. This indicates that the model is particularly robust and aligns closely with the theoretical structure of perceived health threats and beliefs.

Finally, Table 3 shows that the reliability results were adequate, taking into account that they are reliable with values greater than 0.50. The reliability of the Mpox knowledge questionnaire was adequate (KR20=0.70 and α=0.73). The reliability of the questionnaire on Mpox was adequate for each of the dimensions of the instrument, with the perception of threat to health from other contagious viral diseases being the one with the greatest response (α=0.88 and Ω=0.89).

**Table 3.** Indicators of goodness of fit of the confirmatory factor analysis and reliability for the instruments of knowledge, perception and beliefs about Mpox and diseases transmitted through viruses.

	Knowledge	Perception and beliefs		
	D1	D1	D2	D3
**Chi-square of Adjustment (χ ^2^)**	45.21	366.66		
**TLI**	0.92	0.97		
**RMSEA**	0.065 [0.045-0.071]	0.077 [0.065-0.091]		
**SRMR**	0.075	0.074		
**α**	0.73	0.83	0.88	0.81
**KR-20**	0.7	NA	NA	NA
**Ω**	NA	0.83	0.89	0.8

NA: Not applicable.

## 4. Discussion

The second decade of the 21st century has been characterized by the challenge of offering efficient health responses to emerging conditions that involve changes in lifestyles. In the face of infectious conditions such as Mpox, there is a growing need for tools that account for underlying factors that allow us to understand the complexity of the phenomenon and thus guide decision-making in areas such as public health and clinical practice. This perspective, according to the National Institute of Health of Peru, requires coordinated actions that link surveillance and risk assessment approaches and involve actors from society, the media, and academia. ^29^

Based on formative strategies in the Latin American context, the reemergence of infectious conditions leads to the implementation of expected learning outcomes that link epidemiological surveillance processes, the incorporation of sociocultural characteristics analysis in formal research processes in clinical and public health settings. It is crucial that these formative strategies include the development of competencies in data management and interdisciplinary response, allowing health professionals to adapt to the changing dynamics of emerging and reemerging infectious diseases. ^29^

Furthermore, it is essential to promote innovative pedagogical approaches that integrate research with practice, facilitating the creation of solutions adapted to the available resources and the sociocultural particularities of the region. Collaboration between educational institutions, health organizations, and local communities is essential to strengthen the response capacity to health emergencies, thereby improving the resilience of health systems in Latin America. Previous studies have discussed the importance of emerging infectious diseases and their impact on public health, highlighting the need for robust epidemiological surveillance and the adaptation of public health strategies, ^30^ evidencing the challenges these diseases represent in the region and the importance of interdisciplinary research and response. ^31^

The research allowed us to establish that the instrument subjected to the validation process in a sample of Peruvian students retains a similar factor structure based on the characteristics of the original instrument used by Ricco *et al.* (2018; 2022) in a sample of Italian physicians. ^20^ The two factors incorporated in the instrument were characterized by offering information related to knowledge through a unidimensional configuration, while in the second factor it was possible to identify a configuration based on three dimensions. ^32^^–^^34^ In both cases, when performing the reliability analysis using Cronbach’s alpha, Omega and KR 20 criteria, it was evident that the factors were highly reliable with values greater than 0.70. ^32^^,^^34^

In the local context, no similar studies were identified that used this instrument; however, in a validation experience carried out by Yupari-Azabache *et al*., where knowledge, attitudes and practices were measured, a factor structure comprised of four dimensions was reported for knowledge, three dimensions for attitudes and two dimensions for practices, with optimal reliability criteria (Cronbach α between 0.81 and 0.91; McDonald ω between 0.82 and 0.92), similar to those of the present one. ^22^

The study carried out by Caycho -Rodríguez *et al*., established a strategy for the development and initial validation of a scale of fear of Mpox, a dimension that could be considered a *proxy variable* for the attitude factor of this study. The authors reported scale reliability criteria based on a psychological dimension (ω McDonald=0.91) and another affective dimension (ω McDonald=0.71). Additionally, they reported high values of goodness of fit, reliability, and invariance when making comparisons between men (CFI=.99; TLI=.99; RMSEA=.066 [90%CI .004–.111]) and women (CFI=.99; TLI=.99; RMSEA=.067 [90% CI.032–.101]). ^35^

The approach to this zoonotic disease raises the need to incorporate a complex perspective that links knowledge of aspects such as infection mechanisms, close contact with people with the disease, topical lesions, vulnerable groups and the need for vaccination. The above shows the gap in existing knowledge and the needs of the country when considering the experience of the COVID-19 health emergency and the low levels of knowledge reported in recent studies. ^17^^,^^36^

The items related to attitudes suggest the existence of various manifestations that involve the ability to determine the impact on the reality of the country, which corresponds to a macro-systemic perspective towards a transition that involves daily life ^22^ and includes academic work. The above is based on the need to close gaps in knowledge by health professionals, including those in training, and thus improve communication in care processes with a preventive scope.

A study on the level of advanced knowledge and practices in the general population developed in Peru concluded on the need to guarantee the use of validated instruments that respond to sociodemographic and occupational characteristics ^37^; however, given the conditions in which the investigations were developed, the results obtained give an account of the criteria to deepen the mechanisms of situational analysis that involve, in addition to the natural history of the disease, the incubation periods, geospatial characteristics of the most frequent regions, and strategies to avoid the national health emergency. ^17^ Studies in Europe, North America and some in Latin America reported similar needs and raised conditions that require valid mechanisms to approximate a reliable measure in processes of estimating the occurrence of problems associated with the presence of infectious diseases, considering the high emotional burden and the need to promote decision-making based on evidence related to the experience of providing health services, including academic practice settings. ^25^^,^^38^^–^^43^

In the last two decades, Latin American universities have incorporated support strategies based on the allocation of their own resources (technical, human, and financial) and the dissemination of external calls for international cooperation to achieve high standards in research indicators framed within faculty development. Countries such as Brazil, Argentina, Chile, and Mexico stand out for the projection of research results in the consolidation of these initiatives. In the case of countries comparable to Peru, such as Ecuador and Colombia, it has been documented that one of the main limitations is financial support, a condition that affects the degree of scientific production.

To improve faculty participation in research, universities can implement incentives such as reducing teaching loads for those who advise students on research projects, granting recognitions and awards for research excellence, and offering continuous professional development opportunities in research methodologies. ^44^ Additionally, it is essential to foster a research culture from the early years of university education, integrating research into the curriculum and providing access to resources such as scientific journals and academic databases. ^45^ In this regard, educational institutions can adopt concrete measures such as creating more research opportunities through internal and external funding programs, improving research infrastructures, and promoting interdisciplinary and international collaborations. ^46^

Considering the results of this research, it is possible to affirm that professionals in medicine and health sciences need to develop capacities for the administration of instruments and protocols that meet criteria of validity, precision, sensitivity, and specificity. This directly impacts decision-making, the improvement of care quality, and the ability to detect variations in conditions based on interventions according to local needs.

Additionally, it is essential for these professionals to stay updated with the latest research and technological advances in the health field, which will allow them to apply diagnostic and therapeutic tools more effectively. Continuous training and evidence-based education are essential to ensure that health professionals can adapt to the changes and challenges presented by emerging and re-emerging infectious diseases. ^47^

The implementation of specific training programs in validation studies and the promotion of a research culture within health institutions are key strategies to strengthen these capacities. This not only improves the quality of care and patient safety but also fosters a collaborative and multidisciplinary work environment where best practices based on scientific evidence are valued and applied.

The results of this study should be analysed with caution. Since variables such as infectious conditions in the last year, socioeconomic status, or ethnic background were not considered, it is not possible to establish the potential confounding effect. Additionally, it is necessary to consider that the analysis plan did not consider criteria of temporal stability and invariance of the factors based on criteria such as gender, age or academic program, conditions that would increase the scope of establishing the psychometric properties of the instrument.

Regarding the way the sample was obtained, there may be an influence of selection and participation biases in the study. However, given the study’s focus on the psychometric characteristics of the instrument, the obtained sample adequacy index (KMO) supports the baseline generated to provide evidence for factor analysis for the Peruvian and Latin American context.

## 5. Conclusions

The evaluation of knowledge, perceptions and beliefs about the health threats of infectious diseases such as Mpox, becomes relevant in all areas of daily life, including university education. Having instruments that accurately reflect the knowledge, perception and beliefs of health students will make it possible to contribute significantly to the prevention, control and management of this disease and, at the same time, be prepared to address other challenges of public health in the future.

The exploratory scope of this study creates scenarios to consolidate the research line. For future studies, it is expected to estimate indicators of sensitivity to change and determine the concordance between the diagnostic capabilities of students and the criteria of the instructor in clinical settings.

### Ethics and consent

The students who participated in the study did so voluntarily and gave informed consent. The research considered the Declaration of Helsinki, and the protocol was approved by the ethics committee of the Norbert Wiener University, file 1787-2022 dated October 2022 and where it has been considered as a minimum risk research.

## Data Availability

Zenodo: Psychometric Properties of an Instrument Measuring Mon-keypox Knowledge, Perception, and Beliefs of Health Threat in Health Science Students in a Middle-Income Country [Data set]. Zenodo. https://doi.org/10.5281/zenodo.10149774. ^43^ Data are available under the terms of the Creative Commons Attribution 4.0 International license (CC-BY 4.0).

## References

[1] Sklenovská N Van Ranst M : Emergence of Monkeypox as the Most Important Orthopoxvirus Infection in Humans. *Front. Public Health.* 2018;6. 30234087 10.3389/fpubh.2018.00241 PMC6131633

[2] Martín Martín S Morató Agustí M Javierre Miranda A : Prevención de las enfermedades infecciosas. Actualización en vacunas PAPPS 2022. *Aten. Primaria.* 2022;54:102462. 36435581 10.1016/j.aprim.2022.102462 PMC9682159

[3] Centers for Disease Control and Prevention: *cartographer 2022-2023 Mpox Outbreak Global Map.* Washington. D.C:2022.

[4] World Health Organization (WHO): WHO Director-General declares the ongoing monkeypox outbreak a Public Health Emergency of International Concern 2022. Reference Source

[5] Reda A Dhama K : Mpox impact on different organ systems: Complications, mechanisms, and management. *Int. J. Emerg. Med.* 2023;33(4):1–13. 10.1002/rmv.2443 36998233

[6] Petersen E Kantele A Koopmans M : Human Monkeypox: Epidemiologic and Clinical Characteristics, Diagnosis, and Prevention. *Infect. Dis. Clin. N. Am.* 2019;33(4):1027–1043. 30981594 10.1016/j.idc.2019.03.001 PMC9533922

[7] Du M Sun H Zhang S : Global Epidemiological Features of Human Monkeypox Cases and Their Associations With Social-Economic Level and International Travel Arrivals: A Systematic Review and Ecological Study. *Int. J. Public Health.* 2023;68:1605426. 36743344 10.3389/ijph.2023.1605426 PMC9894882

[8] Sharma A Prasad H Kaeley N : Monkeypox epidemiology, clinical presentation, and transmission: a systematic review. *Int. J. Emerg. Med.* 2023;16(1):20. 36932335 10.1186/s12245-023-00491-3 PMC10021050

[9] Huang YA Howard-Jones AR Durrani S : Monkeypox: A clinical update for paediatricians. *J. Paediatr. Child Health.* 2022;58(9):1532–1538. 35979896 10.1111/jpc.16171 PMC9545589

[10] De la Rosa RJG Hernandez JPR : Una nueva alerta mundial en salud causada por la viruela del simio. *Interdiscip. J. Epidemiol. Public Health.* 2022;5(2).

[11] Ramírez-Soto MC : Monkeypox Outbreak in Peru. *Medicina.* 2023;59(6):1096. 37374300 10.3390/medicina59061096 PMC10302731

[12] Al-kassab-Córdova A Ulloque-Badaracco JR Benites-Zapata VA : *Facing Mpox (Former Monkeypox) in Latin America: The Example of Peru and Its Vulnerable Healthcare System.* MDPI;2022;10. 10.3390/vaccines11010010 PMC9863898 36679855

[13] The Lancet Regional H-A: The cycle of neglect: the mpox emergency in the Americas is far from ending. *Lancet Reg. Health Am.* 2023;17:100429. 10.1016/j.lana.2023.100429 PMC9852680 36694820

[14] Stumbar SE Nusynowitz J Jamneshan L : The Need for Emerging Diseases Curricula: A Case Study of Mpox. *PRiMER(Leawood).* 6. 10.22454/PRiMER.2022.922238 PMC9829001 36632498

[15] Martin A Blom IM Whyatt G : A Rapid Systematic Review Exploring the Involvement of Medical Students in Pandemics and Other Global Health Emergencies. *Disaster Med. Public Health Prep.* 2022;16(1):360–372. 32873349 10.1017/dmp.2020.315 PMC7550875

[16] Shi A El Haddad J Cai P : Mpox (monkeypox) information on TikTok: analysis of quality and audience engagement. *BMJ Glob. Health.* 2023;8(3):e011138. 36918216 10.1136/bmjgh-2022-011138 PMC10016284

[17] Regalado Chamorro M Hurtado Letona L Puma Quispe L : Viruela del mono: a un paso de que la historia se repita. *Atención Primaria Práctica.* 2022;4(4):100151. 10.1016/j.appr.2022.100151

[18] Jamil H Idrees M Idrees K : Socio-demographic determinants of monkeypox virus preventive behavior: a cross-sectional study in Pakistan. *BioRxiv.* 2022. 10.1101/2022.12.20.521248 PMC10414588 37561764

[19] Mohamed M Islam M Ahmed S : Assessment of knowledge, attitude, anxiety level and perceived mental healthcare needs toward mpox infection among nursing students: A multi-center cross-sectional study. *Glob. Transit.* 2024;6:203–211. 10.1016/j.glt.2024.10.001

[20] Riccò M Ferraro P Camisa V : When a Neglected Tropical Disease Goes Global: Knowledge, Attitudes and Practices of Italian Physicians towards Monkeypox, Preliminary Results. *Trop. Med. Infect. Dis.* 2022;7(7). 10.3390/tropicalmed7070135 PMC9316880 35878146

[21] Ahmed SK Abdulqadir SO Omar RM : Knowledge, attitude and worry in the kurdistan region of Iraq during the mpox (monkeypox) outbreak in 2022: an online cross-sectional study. *Vaccines.* 2023;11(3):610. 36992194 10.3390/vaccines11030610 PMC10054073

[22] Yupari-Azabache IL Díaz-Ortega JL Bardales-Aguirre LB : Validity and Reliability of the Knowledge, Attitudes and Practices Instrument Regarding Monkey Pox in Peru. *Risk Manag. Healthc. Policy.* 2023;16:1509–1520. 37602360 10.2147/RMHP.S420330 PMC10439795

[23] Lira M Caballero E : Adaptación transcultural de instrumentos de evaluación en salud: Historia y reflexiones del por qué, cómo y cuándo. *Rev. Méd. Clín. Las. Condes.* 2020;31(1):85–94. 10.1016/j.rmclc.2019.08.003

[24] Browne MW : Asymptotically distribution-free methods for the analysis of covariance structures. *Br. J. Math. Stat. Psychol.* 1984;37(1):62–83. 6733054 10.1111/j.2044-8317.1984.tb00789.x

[25] Satorra A Bentler PM Corrections to test statistics and standard errors in covariance structure analysis. In Eye A von Clogg CC , editors. Latent variables analysis: Applications for developmental research. Sage;1994; pp.399–419.

[26] Flora DB Curran PJ : An empirical evaluation of alternative methods of estimation for confirmatory factor analysis with ordinal data. *Psychol. Methods.* 2004;9(4):466–491. 15598100 10.1037/1082-989X.9.4.466 PMC3153362

[27] Olsson U : Maximum likelihood estimation of the polychoric correlation coefficient. *Psychometrika.* 1979;44(4):443–460. 10.1007/BF02296207

[28] Brown TA : *Confirmatory factor analysis for applied research.* Guilford Publications;2015.

[29] Suárez V Cabezas C : Amenazas a la salud pública en el Perú y la respuesta del Instituto Nacional de Salud. *Rev. Peru Med. Exp. Salud Publica.* 2022;39:140–142. 36477313 10.17843/rpmesp.2022.392.11906 PMC11397592

[30] Losa JE : Enfermedades infecciosas emergentes: una realidad asistencial. Anales Sis San Navarra [Internet]. 2021Ago [citado 2024 Nov 21];44(2):147–151. 10.23938/assn.968 Reference Source

[31] Alpuche-Aranda CM : Infecciones emergentes, el gran reto de la salud global: Covid-19. *Salud Pública Méx [Internet].* 2020 Mar-Abr [citado 2024 Nov 21];62(2). 10.21149/11284 32237554

[32] Raykov T Hancock GR : Examining change in maximal reliability for multiple-component measuring instruments. *Br. J. Math. Stat. Psychol.* 2005;58(Pt 1):65–82. 15969840 10.1348/000711005X38753

[33] Trizano-Hermosilla I Alvarado JM : Best Alternatives to Cronbach’s Alpha Reliability in Realistic Conditions: Congeneric and Asymmetrical Measurements. *Front. Psychol.* 2016;7:769. 10.3389/fpsyg.2016.00769 PMC4880791 27303333

[34] Streiner D Norman GR Cairney J : *Health measurement scales: a practical guide to their development and use.* Aust NZJ Public Health;2016. 10.1111/1753-6405.12484 27242256

[35] Caycho-Rodríguez T Vilca LW Carbajal-León C : The Monkeypox Fear Scale: development and initial validation in a Peruvian sample. *BMC Psychol.* 2022;10(1):280. 36443889 10.1186/s40359-022-00997-0 PMC9702644

[36] Navarrete-Mejía PJ Velasco-Guerrero JC Sullcahuamán-Valdiglesias E : Conocimiento sobre viruela del mono en profesionales de la salud, Lima-Perú. *Revista del Cuerpo Médico Hospital Nacional Almanzor Aguinaga Asenjo.* 2022;15:252–255. 10.35434/rcmhnaaa.2022.152.1547

[37] Mamani-Benito O Carranza Esteban R Fernández J : Adaptation and Validation of a Monkeypox Concern Instrument in Peruvian Adults. *Sustainability.* 2022;14:12354. 10.3390/su141912354

[38] Patiño LH Guerra S Muñoz M : Phylogenetic landscape of Monkeypox Virus (MPV) during the early outbreak in New York City, 2022. *Emerg. Microbes Infect.* 2023;12(1):e2192830. 36927408 10.1080/22221751.2023.2192830 PMC10114986

[39] Chakraborty C Bhattacharya M Saikumar G : A European perspective of phylogenomics, sublineages, geographical distribution, epidemiology, and mutational landscape of mpox virus: Emergence pattern may help to fight the next public health emergency in Europe. *J. Infect. Public Health.* 2023;16(7):1004–1014. 37172461 10.1016/j.jiph.2023.04.017 PMC10147450

[40] Hatmal MM Al-Hatamleh MAI Olaimat AN : Comprehensive literature review of monkeypox. *Emerg. Microbes Infect.* 2022;11(1):2600–2631. 36263798 10.1080/22221751.2022.2132882 PMC9627636

[41] Huaire Inacio EJ Herrera Álvarez AM Sifuentes De la Cruz LE : Retorno a la presencialidad: Actitudes de los universitarios peruanos hacia el aprendizaje y pos-crisis sanitaria. *Revista de Ciencias Sociales.* 2023;13159518:29.

[42] Carreño-Moreno SP Chaparro-Díaz L Cifuentes-Tinjaca CD : Conocimientos, actitudes, prácticas, temor y estrés ante el Covid-19 en estudiantes y recién egresados de Enfermería en Colombia. *Revista Cuidarte.* 2021;12(3). 10.15649/cuidarte.2044 PMC11290800 40115236

[43] Rivera-Lozada O Rivera Lozada IC Castro-Alzate ES : Psychometric Properties of an Instrument Measuring Mon-keypox Knowledge, Perception, and Beliefs of Health Threat in Health Science Students in a Middle-Income Country.[Data set]. *Zenodo.* 2023. 10.5281/zenodo.10149774

[44] Cervantes E : Un Acercamiento a la Formación de Docentes como Investigadores Educativos en México. *REICE [Internet].* 2019[citado 2024 Nov 21];17(4):59–74. 10.15366/reice2019.17.4.003 Reference Source

[45] Palencia Salas V Verdugo PA : La investigación como responsabilidad docente. Un análisis de las políticas educativas en Colombia y Chile. *Pág. Educ. [Internet].* 2023[citado 2024 Nov 20];16(1): e3060. 10.22235/pe.v16i1.3060 Reference Source

[46] Blanco-Portela N Poza-Vilches MF Junyent-Pubill M : Estrategia de investigación-acción participativa para el desarrollo profesional del profesorado universitario en educación para la sostenibilidad: “Academy sustainability Latinoamérica” (ACSULA). *Prof. Rev. Curric. Form. Profr.* 2020;24(3):99–123. 10.30827/profesorado.v24i3.15555

[47] Arias Capote JC Hidalgo Mesa CJ Hidalgo Cepero HE : Utilidad de las guías, protocolos y algoritmos en la práctica clínica. *Rev. Cubana. Med [Internet].* 2019 Ene-Mar [citado 2024 Nov 21];58(1). Epub 20-Feb-2020. Reference Source

