# Supplementary material for: Psychometric properties of an instrument measuring monkeypox knowledge, perception, and beliefs of health threat in health science students in a middle-income country
Source: F1000Res. 2026 Aug 4;13:627. Originally published 2024 Jun 12. [Version 3] doi: 10.12688/f1000research.145169.3 (PMC13736061; doi:10.12688/f1000research.145169.3)
Supplement: Peer review history

# Reviewer report: Reviewer response for version 3

**DOI:** 10.5256/f1000research.196703.r531878

**Published:** 2026-11-04

**Authors:** Patrick M. Mutua

**Affiliations:** 1 Pwani University, Kilifi, Kenya

The manuscript addresses an important and relevant public health issue by evaluating the psychometric properties of an instrument for assessing Mpox knowledge, perceptions, and health threat beliefs among health sciences students in Peru. The study has potential value in supporting the development of reliable assessment tools for emerging infectious disease preparedness. The use of exploratory and confirmatory factor analyses, together with multiple reliability coefficients, is a methodological strength.

 However, several issues require attention before the manuscript can be considered for indexing:

 1. Methodological clarity: The authors should clarify the factor structure of the instrument, particularly how the three dimensions of the perception and health threat beliefs construct were identified and confirmed. The distinction between exploratory and confirmatory factor analysis also needs clearer reporting.

 2. Sampling and generalizability: The use of convenience sampling and recruitment from only two universities, with approximately 91% of participants drawn from private universities, limits the representativeness of the sample. This limitation should be more explicitly reflected in the conclusions.

 3. Psychometric reporting: The authors should provide a more comprehensive presentation of factor loadings, eigenvalues, explained variance, and model fit statistics for each construct. Additionally, the justification for considering reliability coefficients above 0.50 acceptable requires reconsideration, as this threshold may be too permissive for an instrument intended for research applications.

 4. Instrument adaptation: Although translation and back-translation are reported, the authors should provide further details on the pilot testing and cultural adaptation procedures, including how the suitability and relevance of individual items were established.

 5. Discussion and conclusions: The discussion contains considerable general commentary on research capacity and university research development that is not directly supported by the study findings. It would benefit from greater focus on the psychometric findings, their implications, and the limitations of the instrument validation process.

Is the work clearly and accurately presented and does it cite the current literature?

Yes

If applicable, is the statistical analysis and its interpretation appropriate?

Partly

Are all the source data underlying the results available to ensure full reproducibility?

Partly

Is the study design appropriate and is the work technically sound?

Partly

Are the conclusions drawn adequately supported by the results?

Yes

Are sufficient details of methods and analysis provided to allow replication by others?

No

Reviewer Expertise:

Immunology and Public Health

I confirm that I have read this submission and believe that I have an appropriate level of expertise to confirm that it is of an acceptable scientific standard, however I have significant reservations, as outlined above.

# Reviewer report: Reviewer response for version 2

**DOI:** 10.5256/f1000research.175950.r405377

**Published:** 2025-08-18

**Authors:** James Andrawus, Kefas Bitrus

**Affiliations:** 1 Federal University Dutse, Dutse, Jigawa, Nigeria; 2 Mathematics, Federal University Lokoja (Ringgold ID: 487347), Lokoja, Kogi, Nigeria

The article is sound, and the following are the minor comments on this paper that should be carefully addressed in the revision: 

 1. In the abstract, under methods, what is the instrument made by Ricco et al.?  The authors need to describe this method using a few words

 2. In the abstract, under results, the authors need to explicitly write the implications of when " RMSEA and SRMR coefficients lower than 0.08 and a TLI lower than 0.90"

 3.  In the abstract, under conclusions, the authors are supposed to give a scientific conclusion.

 4. In subsection 2.5, the authors mentioned "the weighted least squares means and variance-adjusted estimation (WLMSV) and polychoric matrix, as well as oblique rotation (quatrimin)" but no statistical or mathematical formula is written there to validate their statement.

 5. This statement, "Additionally, it included the calculation of explained variance to understand the total variation in the data explained by the factors identified in the exploratory factor analysis," needs to be backed with a formula to validate the statement.

Is the work clearly and accurately presented and does it cite the current literature?

Yes

If applicable, is the statistical analysis and its interpretation appropriate?

Yes

Are all the source data underlying the results available to ensure full reproducibility?

Yes

Is the study design appropriate and is the work technically sound?

Yes

Are the conclusions drawn adequately supported by the results?

Yes

Are sufficient details of methods and analysis provided to allow replication by others?

Partly

Reviewer Expertise:

Mathematical Epidemiology

We confirm that we have read this submission and believe that we have an appropriate level of expertise to confirm that it is of an acceptable scientific standard.

# Response

**Published:** 2025-08-24

**Authors:** ORIANA RIVERA LOZADA

**Affiliations:** Universidad Nacional Mayor de San Marcos, Lima District, Lima Region, Peru

**Response to Reviewer Comments**

**Reviewer Comment:**

 1. In the abstract, under methods, what is the instrument made by Ricco et al.? The authors need to describe this method using a few words.

**Author Response:**

 We have added a brief description of the instrument developed by Ricco et al: “The study variable on knowledge of Mpox was assessed using a 17-item questionnaire developed by Ricco et al., originally applied to health professionals, adapted to Peruvian context, and administered to 416 students.”

**Reviewer Comment:**

 2. In the abstract, under results, the authors need to explicitly write the implications of when 'RMSEA and SRMR coefficients lower than 0.08 and a TLI lower than 0.90'.

**Author Response:**

 We have revised the results section of the abstract to explicitly state that RMSEA and SRMR coefficients below 0.08 indicate acceptable model fit, whereas a TLI value below 0.90 suggests that the model does not reach the threshold for adequate fit. This clarification has been added to improve interpretability.

 “The results showed adequate goodness-of-fit indicators, with RMSEA and SRMR values below 0.08 and a TLI above 0.90, confirming an acceptable model fit. Reliability was adequate for knowledge of Mpox (KR20 = 0.70 and α = 0.73), with perception of health threats showing the highest reliability (α = 0.88 and Ω = 0.89).”

**Reviewer Comment:**

 3. In the abstract, under conclusions, the authors are supposed to give a scientific conclusion.

**Author Response:**

 We have revised the conclusion of the abstract to provide a clear scientific statement. It now emphasizes that the validated instrument demonstrates robust psychometric properties and is suitable for assessing attitudes in the target population, contributing to the scientific understanding and monitoring of the studied construct.

 ”The instrument demonstrated an adequate factor structure and acceptable reliability for measuring Mpox knowledge, perception, and health threat beliefs in Peruvian health students, supporting its applicability in future research and public health interventions.”

**Reviewer Comment:**

 4. In subsection 2.5, the authors mentioned 'the weighted least squares means and variance-adjusted estimation (WLSMV) and polychoric matrix, as well as oblique rotation (quatrimin)' but no statistical or mathematical formula is written there to validate their statement.

**Author Response:**

 We have addressed this by including the mathematical formulation for the weighted least squares means and variance-adjusted (WLSMV) estimator in subsection 2.5, as well as clarifying its relation with the polychoric correlation matrix and oblique (quatrimin) rotation. This addition provides the required statistical justification.

 “This estimator minimises the discrepancy between the sample covariance matrix (��) and the model-implied covariance matrix (��(��)) as a function of the parameters ��:

*F* *WLMSV* *=(s-*σθ)' *W* *-1* *(s-*σθ) 

 Where W is the inverse of the asymptotic covariance matrix of �� ^24^ . WLSMV employs robust standard errors and a mean–variance adjusted ��2 test statistic ^2^ ^5-26^ . For ordinal variables, polychoric correlations were computed, defined as the Pearson correlation between continuous latent variables underlying the observed ordinal responses ^26-27^ . Finally, an oblique quartimin rotation was applied to enhance factor interpretability.”

**Reviewer Comment:**

 5. This statement, 'Additionally, it included the calculation of explained variance to understand the total variation in the data explained by the factors identified in the exploratory factor analysis,'

**Author Response:**

 We have expanded this section to clarify how explained variance was calculated. Specifically, it was defined as the ratio of the sum of the eigenvalues of the retained factors to the total eigenvalues, expressed as a percentage. Consistent with psychometric standards, values above 60% are considered acceptable (Brown, 2015).

 “Additionally, the explained variance was calculated as the ratio of the sum of the eigenvalues of the retained factors to the total eigenvalues, expressed as a percentage. Values above 60% are generally considered acceptable in psychometric studies”

**Response to Reviewer Comments**

**Reviewer Comment:**

 1. In the abstract, under methods, what is the instrument made by Ricco et al.? The authors need to describe this method using a few words.

**Author Response:**

 We have added a brief description of the instrument developed by Ricco et al: “The study variable on knowledge of Mpox was assessed using a 17-item questionnaire developed by Ricco et al., originally applied to health professionals, adapted to Peruvian context, and administered to 416 students.”

**Reviewer Comment:**

 2. In the abstract, under results, the authors need to explicitly write the implications of when 'RMSEA and SRMR coefficients lower than 0.08 and a TLI lower than 0.90'.

**Author Response:**

 We have revised the results section of the abstract to explicitly state that RMSEA and SRMR coefficients below 0.08 indicate acceptable model fit, whereas a TLI value below 0.90 suggests that the model does not reach the threshold for adequate fit. This clarification has been added to improve interpretability.

 “The results showed adequate goodness-of-fit indicators, with RMSEA and SRMR values below 0.08 and a TLI above 0.90, confirming an acceptable model fit. Reliability was adequate for knowledge of Mpox (KR20 = 0.70 and α = 0.73), with perception of health threats showing the highest reliability (α = 0.88 and Ω = 0.89).”

**Reviewer Comment:**

 3. In the abstract, under conclusions, the authors are supposed to give a scientific conclusion.

**Author Response:**

 We have revised the conclusion of the abstract to provide a clear scientific statement. It now emphasizes that the validated instrument demonstrates robust psychometric properties and is suitable for assessing attitudes in the target population, contributing to the scientific understanding and monitoring of the studied construct.

 ”The instrument demonstrated an adequate factor structure and acceptable reliability for measuring Mpox knowledge, perception, and health threat beliefs in Peruvian health students, supporting its applicability in future research and public health interventions.”

**Reviewer Comment:**

 4. In subsection 2.5, the authors mentioned 'the weighted least squares means and variance-adjusted estimation (WLSMV) and polychoric matrix, as well as oblique rotation (quatrimin)' but no statistical or mathematical formula is written there to validate their statement.

**Author Response:**

 We have addressed this by including the mathematical formulation for the weighted least squares means and variance-adjusted (WLSMV) estimator in subsection 2.5, as well as clarifying its relation with the polychoric correlation matrix and oblique (quatrimin) rotation. This addition provides the required statistical justification.

 “This estimator minimises the discrepancy between the sample covariance matrix (��) and the model-implied covariance matrix (��(��)) as a function of the parameters ��:

*F* *WLMSV* *=(s-*σθ)' *W* *-1* *(s-*σθ) 

 Where W is the inverse of the asymptotic covariance matrix of �� ^24^ . WLSMV employs robust standard errors and a mean–variance adjusted ��2 test statistic ^2^ ^5-26^ . For ordinal variables, polychoric correlations were computed, defined as the Pearson correlation between continuous latent variables underlying the observed ordinal responses ^26-27^ . Finally, an oblique quartimin rotation was applied to enhance factor interpretability.”

**Reviewer Comment:**

 5. This statement, 'Additionally, it included the calculation of explained variance to understand the total variation in the data explained by the factors identified in the exploratory factor analysis,'

**Author Response:**

 We have expanded this section to clarify how explained variance was calculated. Specifically, it was defined as the ratio of the sum of the eigenvalues of the retained factors to the total eigenvalues, expressed as a percentage. Consistent with psychometric standards, values above 60% are considered acceptable (Brown, 2015).

 “Additionally, the explained variance was calculated as the ratio of the sum of the eigenvalues of the retained factors to the total eigenvalues, expressed as a percentage. Values above 60% are generally considered acceptable in psychometric studies”

# Reviewer report: Reviewer response for version 1

**DOI:** 10.5256/f1000research.159078.r338479

**Published:** 2024-11-14

**Authors:** Mona Gamal Mohamed

**Affiliations:** 1 RAK Medical and Health Sciences University, Ras Al Khaimah, United Arab Emirates

**Introduction:**

 The introduction lacks a comprehensive discussion of the psychometric framework and the significance of the study. While the disease context is briefly mentioned, the foundational importance of this work in advancing measurement science is not adequately addressed. Contrary to the claim of limited research in this area, a substantial body of literature exists, providing critical insights into similar psychometric developments. These references should be acknowledged to contextualize the study and highlight its relevance within the broader research landscape.

**Methods:**

 The methodology section is underdeveloped and lacks clarity, making it difficult to follow the process. Only one reference is cited for the instrument's development, despite the wealth of existing literature on similar psychometric tools, which I have included in the included citations. Furthermore, the translation process is entirely omitted, and the critical phases of tool validation—such as content validation, construct validation, and reliability testing—are not detailed. These omissions significantly undermine the credibility and reproducibility of the study.

**Results:**

 The results section requires substantial improvement through advanced statistical analysis. The current presentation appears overly simplistic, offering only a fundamental analysis that does not provide a nuanced understanding of the tool's psychometric properties. Enhanced analysis is necessary to give the reader a clearer and more comprehensive perspective on the validity and reliability of the instrument. Such improvements would significantly elevate the quality and usefulness of the findings.

Is the work clearly and accurately presented and does it cite the current literature?

Partly

If applicable, is the statistical analysis and its interpretation appropriate?

No

Are all the source data underlying the results available to ensure full reproducibility?

Partly

Is the study design appropriate and is the work technically sound?

No

Are the conclusions drawn adequately supported by the results?

Partly

Are sufficient details of methods and analysis provided to allow replication by others?

No

Reviewer Expertise:

Infectious disease , Meta-analysis, qualitative and quantitative research

I confirm that I have read this submission and believe that I have an appropriate level of expertise to state that I do not consider it to be of an acceptable scientific standard, for reasons outlined above.

## References

[1] : The 2022 monkeypox outbreak and associated psychiatric morbidities. *International Journal of Surgery* .2022;106: 10.1016/j.ijsu.2022.106913 10.1016/j.ijsu.2022.106913 PMC953382536113838

[2] : Assessment of knowledge, attitude, anxiety level and perceived mental healthcare needs toward mpox infection among nursing students: A multi-center cross-sectional study. *Global Transitions* .2024;6: 10.1016/j.glt.2024.10.001203-211 10.1016/j.glt.2024.10.001

[3] : Monkeypox virus infection and myocarditis: A review of current evidence and possible pathogenesis. *Narra J* .2023;3(1) : 10.52225/narra.v3i1.104e104 38450040 10.52225/narra.v3i1.104 PMC10914025

# Reviewer report: Reviewer response for version 1

**DOI:** 10.5256/f1000research.159078.r323813

**Published:** 2024-10-09

**Authors:** Alysa Pomer

**Affiliations:** 1 Center for Surgery and Public Health, Brigham and Women’s Hospital, Boston, MA, USA

**Summary:**

 In this study, the authors seek to validate a psychometric instrument measuring mpox knowledge, perceptions, and beliefs of health threats in a new context—namely, among healthcare students at two universities in Peru. This is a valuable contribution in Peru (and potentially other Spanish-speaking populations of the Americas) where mpox rates have been high. Understanding the knowledge base and biases of medical trainees around this disease can help ensure education of those future medical providers is adapted so mpox patients are getting the best care possible.

 The methods should be expanded to ensure replication is possible. This should include information about the specific items included as well as the translation procedures.

 The specific statistical analyses used are outside of my area of familiarity, so I can’t comment on the statistical analyses specifically. However, some information does seem to be missing from the tables that would have made it easier for me to understand these analyses.

 My greatest concern with this manuscript is that the specific results of this study are not clearly shown in the Results, which means the Discussion does not have a thorough exploration of what this particular study found in relation to other studies, leading to a vague Conclusion that doesn’t say anything specific about this study. If the Results can be more clearly and thoroughly explained, I believe this would cascade to a more robust Discussion section and clear, concise Conclusion.

 It is worth noting that the nomenclature around this disease changed fairly recently; I strongly suggest changing “monkeypox” to “mpox” throughout the manuscript. The literature is otherwise current.

 Finally, the manuscript would benefit from proofreading for grammar and syntax.

**General comments:**

 1. I strongly suggest changing “monkeypox” to “mpox” to align with updated WHO nomenclature guidelines ( https://www.who.int/news/item/28-11-2022-who-recommends-new-name-for-monkeypox-disease and https://www.ncbi.nlm.nih.gov/pmc/articles/PMC9901940/).

**Introduction:**

 2. Update your first sentence to note that “monkeypox” is the previous name for the disease that is currently known as “mpox” and that this disease is caused by the monkeypox virus (MPXV).

 3. Did the last outbreak start in May 2022 in the UK, or was it confined to May 2022 in the UK? The current language makes it sound like the latter, but that’s contradicted in the following line when it’s stated that a PHE was declared two months later. Please clarify.

 4. Compared to 2022…”—is this referring to case rates in 2023 being compared to 2022 case rates? Please clarify.

**Methods:**

 5. Population: I suggest presenting the inclusion criteria rather than the exclusion criteria.

 6. Variables and instruments: “The study variable on knowledge of [mpox] was taken from the instrument…and carried out with health professionals.” How are the health professionals involved with the survey? I understood the survey to be conducted online and given to students. Please clarify.

 7. Variables and instruments: Which 17 knowledge items and 11 perception/belief items were included? Why were these items included but not the others? Consider including the survey used in this study as a supplementary file (Spanish and English translations).

 8. Variables and instruments: Considering the importance of translation in the validation of survey instruments in new contexts, the information about the translation procedure should be expanded. For example, what does it mean that the instrument was “translated by professionals and evaluated by expert judgment”? How many people translated the survey? Were the translators from the medical field? Were any of the translators part of the study team? Did a second set of translators back-translate?

 9. Procedure: “Irrelevant” would be a more accurate word than “impertinent” in the first sentence.

**Results:**

 10. A table showing the knowledge items, correct answer, and response distribution for each item would be helpful (similar to table A3 in the Ricco et al., 2022, paper).

 11. The results of the perception and belief of health threats variables should also be presented in a table.

**Discussion:**

 12. Clarification—If the first sentence is referring to the 2020s, this is actually the third decade of the 21st century (2000s is the first decade, 2010s is the second decade, 2020s is the third decade).

**Conclusions:**

 13. This is a very vague statement that doesn’t say anything about what this particular study actually found. Clarify the findings through the Results and Discussion sections to create a more specific Conclusions section.

**References:**

 14. #14 is an incomplete reference and should be as follows: [ ref 1 ]

**Tables:**

 15. Table 1 should include the total number of participants.

 16. Tables 2 and 3 include “D1, D2, D3” in the header but don’t define what these mean. Please define this terminology as a footnote in the tables and consider adding the information to the text.

 17. Table 3 shows RMSEA with bracketed ranges, but no other indicators show these ranges. Please explain what these ranges are and why the other indicators do not include ranges.

Is the work clearly and accurately presented and does it cite the current literature?

Partly

If applicable, is the statistical analysis and its interpretation appropriate?

I cannot comment. A qualified statistician is required.

Are all the source data underlying the results available to ensure full reproducibility?

Partly

Is the study design appropriate and is the work technically sound?

Yes

Are the conclusions drawn adequately supported by the results?

Partly

Are sufficient details of methods and analysis provided to allow replication by others?

Partly

Reviewer Expertise:

Biological Anthropology, infectious diseases, survey design and research

I confirm that I have read this submission and believe that I have an appropriate level of expertise to confirm that it is of an acceptable scientific standard, however I have significant reservations, as outlined above.

## References

[1] : The Need for Emerging Diseases Curricula: A Case Study of Mpox. *PRiMER* .2022;6: 10.22454/PRiMER.2022.922238922238 36632498 10.22454/PRiMER.2022.922238 PMC9829001
